# Introducing the Bidimensional Model of Hope and its conceptual and methodological utilities

**DOI:** 10.3389/fpsyg.2024.1456303

**Published:** 2024-11-19

**Authors:** Oded Adomi Leshem, Eran Halperin

**Affiliations:** Department of Psychology, Hebrew University of Jerusalem, Jerusalem, Israel

**Keywords:** Bidimensional Model of Hope, hope conceptualization, hope definition, hope measurement, hope in conflict, hope and politics, hope and social change

## Abstract

What is hope, and how can we measure it? These questions have occupied the minds of hope scholars across disciplines. This article outlines a comprehensive approach to understanding hope: the Bidimensional Model of Hope. Building on the standard definition of hope, the bidimensional model explores hope as the intersection between wishes (desires, aspirations) and expectations (assessment of possibility). Hope is thus located on a bidimensional plane with two perpendicular axes; one corresponds to the levels of wishes to achieve an outcome, and the second to the levels of expectations of achieving the outcome. We claim that the bidimensional approach is comprehensive enough to include existing definitions of hope while being parsimonious, versatile, and applicable to many contexts, including those where the hoped-for outcome is beyond people’s actual or perceived control. We show the model’s theoretical and methodological utility and its use in existing qualitative and quantitative research on hope in the context of intractable international conflicts. We end with suggesting pathways for developing and applying the Bidimensional Model of Hope to assist hope research in a variety of contexts and disciplines.

## Introduction

Hope is a fascinating concept. It is a central theme in religious scriptures, countless works of art, and the oratory of political and spiritual leaders. Trying to grasp its complexity and innate elusiveness, philosophers and theologians have offered numerous ways to understand hope, its virtues and shortcomings, and its role in the lives of individuals and collectives (e.g., Bloch, 1959; Fromm, 1968; Moltmann, 1968; Rorty, 1999). In the second half of the 20th century, psychologists joined the effort of defining and conceptualizing hope (e.g., Lopez and Snyder, 2003; Seligman, 1990; Snyder et al., 2005; Stotland, 1969). As the thorough reviews by Callina et al. (2018), Krafft et al. (2023), and Scioli (2020) demonstrate, the scholarly work on hope is vast, impressive, and highly insightful.

The intellectual fascination with hope is well deserved. However, as humankind grapples with the profound uncertainties of the 21st century, hope and its absence are no longer abstract concepts. They are becoming stark realities for many around the world. The mass shifts in populations and environment, the rise of artificial intelligence and global pandemics, and the unprecedented challenges we face on local and global levels may push people into profound uncertainty and hopelessness. In this context, understanding hope and its implications is not just an intellectual pursuit but a pressing need.

The question of hope is certainly relevant for people mired in longstanding intractable conflicts, where the unbearable reality of war is doubled by the likewise unbearable belief that the bloodshed will never end. Can hope emerge amidst war and destruction? If so, can we direct hope to promote peace? As political psychologists studying hope amidst conflict, these questions are fundamental to our research agenda. Yet our interest in hope’s manifestation during conflict has triggered a much deeper exploration of hope in its most basic form, stripped from specific functions and contexts.

Several commonly cited and influential conceptualizations of hope exist, including Snyder’s Hope Theory (Snyder, 2000) that examined how hope operates in the context of goal pursuit and Herth’s hope model (Herth, 1991, 2005) that was initially developed to explore hope among people with severe illness. Herth’s model provided an essential contribution as it explored hope that is not necessarily goal-oriented and could also be located outside of the individual’s control (e.g., hope among the terminally ill).^1^

Snyder’s and Herth’s models provide expansive explanations of the process of hope, how it functions in many contexts, and its intricate network of manifestations and connotations. We, however, were interested in hope in its nucleus state, stripped of functionality and connotations. In other words, we wanted to know what hope “is” before we explored how it “works.” We felt that exploring hope’s bare anatomy and, by that, denoting its most skeletal structure would offer insights into hope relevant not only on the personal level but also on a collective level in social and political contexts and to hope toward things located outside of the individual’s goals and scope of control.

The need to understand hope amid conflict was thus the spark that pushed us to develop a model pertinent to hope across contexts. Merging philosophical (e.g., Day, 1969; Milona, 2020) and psychological (e.g., Staats and Stassen, 1985; Stotland, 1969) insights with lay interpretations of hope (e.g., Bruininks and Malle, 2005; Li et al., 2021), we offer the Bidimensional Model of Hope, which we believe could be relevant to various contexts and functions.

Our research endeavor began almost by accident. More precisely, it started from the need to solve a methodological problem rather than from theoretical or conceptual curiosity. One of our main research programs is to reveal the psychological drivers of conflict in societies locked in prolonged violent disputes. Within this program, we were interested in measuring the levels of hope for peace among Israelis and Palestinians, two peoples mired in decades of hostilities and war. Looking at existing literature on hope in the context of this conflict, we noticed mixed results. Some studies report high levels of hope for peace among participants (Antonovsky and Arian, 1972; Halperin et al., 2008), while others report that levels of hope for peace are extremely low (Rosler et al., 2017; Stone, 1982). Looking more closely at the studies, it seems that when participants reported their levels of “hope” for peace, some reported how much they wished for peace (very much), while others reported how much they expected peace to materialize (not very much). In other words, “hope” was used as a synonym for each one of two distinct concepts: “wishes” and “expectations.” How could we measure peoples’ hope for peace if hope can sometimes mean “wish” and sometimes “expect” (and sometimes a blend of the two)?

As this paper outlines, the bidimensional approach offers a solution to the measurement problem. Perhaps more importantly, what started as an attempt to solve a methodological conundrum developed into a comprehensive model. Simply put, we suggest hope is best understood as a bidimensional mental construct, with one dimension corresponding to the extent one wishes (desires, aspires) for an outcome and the second corresponding to the extent one expects the outcome to transpire. Looking at hope as a construct consisting of wishes and expectations is not entirely new. It is apparent in the “Standard Definition” of hope used in philosophy and was used in earlier work in psychological inquiries into hope (e.g., Erickson et al., 1975; Sagy and Adwan, 2006; Staats and Stassen, 1985; Stotland, 1969). Our humble contribution is to turn existing work that understood hope as comprising wishes and expectations into a model that can be used across contexts, disciplines, and methodologies. What is more, the Bidimensional Model we suggest considers wishes and expectations not as components of a unidimensional concept but as *orthogonal dimensions* that define a two-dimensional space where hope is located.

We and other scholars have already used the bidimensional model to investigate hope (Hasler et al., 2023; Leshem, 2017, 2019, 2023; Leshem et al., 2023a; Leshem et al., 2023b; Ushomirsky et al., 2023; Leshem and Halperin, 2020a, 2020b, 2023; Shani et al., in press; Ushomirsky et al., 2023; Wenzel et al., 2024) but we have not had the opportunity to explicate its wider utility. In this paper, we explain the rationale behind the model, outline its applicability, and compare it to existing models, in particular Snyder’s Hope theory. We also elaborate on why wishes do not aggregate or multiply but are better understood as two perpendicular dimensions forming the hope “space” and demonstrate the theoretical versatility of the model in qualitative and quantitative research on hope.

The remainder of the paper continues as follows. We first provide a brief outline of existing psychological conceptualizations of hope. We then turn to lay interpretations of hope and explain why understanding the everyday use of hope is a crucial resource for hope conceptualization. We then turn to introduce the bidimensional model and its visual representation. We then present the conceptual and methodological utility of the bidimensional approach and provide examples of how the model can clarify hope’s role in various contexts. We end by offering paths for applying the model and future research of the bidimensional approach.

## Existing conceptualizations of hope

As this paper cannot cover the full range of existing conceptualizations of hope, we offer a very concise overview of major trends in hope conceptualizations, focusing on psychological approaches. Following Krafft et al. (2023), it is useful to divide the psychological conceptualization of hope into the following categories.

### Cognitive approaches to hope conceptualization

Perhaps the most-cited cognitive approach to hope is Synder’s Hope Theory (Snyder et al., 2005). According to Snyder, hope is comprised of two components. The first is *pathway thinking*, which happens when the “protagonist perceives themselves as able to produce a route to a goal” (Snyder, 2000, p. 9). The second is *agency*, which “reflects the person’s perception that he or she can begin movement along the imagined pathways to goals” (Snyder, 2000, p. 10). When these two components add up, they enhance each other as the “goal pursuit process unfolds” (Snyder, 2000). Snyder’s hope theory explains how hope plays out when people think about their goals and work to attain them. The pathway-thinking/agency approach can be applied to many types and manifestations of hope. For example, trait hope investigates pathway-thinking and agency as personality dispositions and thus seeks to reveal individual differences in people’s general approaches to their goals (Snyder et al., 1991). State hope, on the other hand, focuses on people’s pathway-thinking and agency at a particular moment (Snyder et al., 1996). Last, domain-specific hope moves the focus of hope from the general to life arenas such as family, work, and leisure (Snyder, 2000). Snyder’s theory is highly valuable because it explains how agency and pathway thinking contribute to and interact in the often challenging process of goal attainment. Indeed, Snyder’s emphasis on pathway thinking and agency has been the dominant cognitive theory of hope (Rand and Rogers, 2023).^2^ However, initial psychological inquiries into hope were based on a somewhat more basic approach. One of the pioneering works on hope as a mental construct was led by Stotland (1969), who mentioned two elements intrinsic to hope. The first was the “organism’s perceived importance of the goal,” and the second was the organism’s “perceived probability of obtaining that goal.” Hope thus depends on how much the goal is evaluated as desirable and probable. Erickson et al. (1975) and Staats (1989) continued Scotland’s approach by separately measuring peoples’ wishes and expectations for an array of pre-defined goals. Additional work in psychology explored hope as the combination of wishes and expectations (e.g., Sagy and Adwan, 2006; Wenzel et al., 2024). However, this approach did not develop into a comprehensive model and historically remained unexplored.

### Conceptualizing hope as an emotion

Understanding hope as an emotion is apparent in the writing of hope philosophers (e.g., Bloch, 1959; Blöser, 2020; van den Heuvel, 2020). Emotion is also the primary lens used by many psychologists (e.g., Lazarus, 1999; van Zomeren et al., 2019). Lazarus’s appraisal theory of emotions (Lazarus, 1999) asserts that emotions emerge from rapid evaluations of situations. The emotion of hope is activated through two quick appraisals: the assessment of the desirability of a target and the evaluation of the chances of attaining it. Like Stotland’s cognitive approach to hope, Lazarus’ emotional perspective also hints at the need to study hope as the intersection between wishes and expectations.

Examining hope as an emotion poses a challenge because emotions are often transient (Halperin, 2016; Itkes and Kron, 2019; Robinson and Clore, 2002a, 2002b). People might experience hope when they learn that their wishes are more likely to come true. However, after some time, the emotional experience fades and eventually disappears. Conceptualizing hope as a sentiment addresses the problem of emotions’ short duration. Sentiments are a “disposition to respond emotionally to a certain object” (Frijda et al., 1991, p. 207). As such, sentiments are long-lived affective experiences and thus correspond better with situations where hope is a prolonged experience. Hope may be defined as an emotion and a sentiment because it is sometimes experienced immediately but can evolve into a lingering disposition if experienced over time (Halperin et al., 2011).

As stated, some hope scholarships classify hope as a cognition (e.g., Cheavens and Whitted, 2023; Snyder, 2000; Stotland, 1969) while others categorize hope as an emotion (e.g., Hasan-Aslih et al., 2020; Lazarus, 1999). Yet, recent work in psychology follows an integrative approach (e.g., Cohen-Chen and Halperin, 2023) that sees cognitive and emotional mental states as intertwined rather than separated. Indeed, looking at hope as a mental state involving emotion and cognition was already proposed by Staats & Stassen, who explored hope as an “affective cognition”. Herth (1991, 2005) has also integrated cognitive and affective aspects in her model. The model divides hope into a cognitive-temporal component related to the perception that a desired outcome is realistically probable and an affective-behavioral component consisting of confidence and an intention to pursue the desired outcome. Herth also adds another component, the affiliative-contextual component related to interconnectedness with others and spirit.

Although limited, the above overview highlights some of the major psychological conceptualizations of hope. It is worth mentioning that these models were based on what scholars think hope is, leaving aside lay interpretations of the concept. Yet, understanding lay interpretations of theoretical concepts is essential when testing scholarly theories in the “real world” (Furnham, 1988). Past studies revealed the problems generated by the gaps between how concepts like “culture” (Lo and Sasaki, 2024) and “race” (Dubriwny et al., 2005) are understood inside and outside academia. Lay interpretations can teach us about the “common-sense” account of the phenomenon while providing a critical lens on scholarly interpretations (Li et al., 2021). Recognizing the differences between how scholars and laypersons interpret concepts is particularly crucial in research relying on self-reported measurements (Leshem and Halperin, 2020c). The soundness of research could be seriously infringed if scholars explore one thing while respondents refer to another.

## Colluqual interpretations of hope

Several projects explored the lay interpretations of hope. Feldman et al. (2023) investigated how healthcare professionals understood the concept of hope. Analysis of data collected from the 176 participants shows that the most mentioned categories associated with hope were “cognition” (97.2%), “implicit goal” (85.8%), “agency thoughts” (45.4%), “future-oriented” (38.6%), “likely” (36.9%), “affect” (33.5%), and “pathway thoughts” (29.5%). The authors point out that lay interpretations are somewhat inconsistent with Snyder’s Theory (less than 30% associated hope with pathway thinking) and Herth’s model (cognition was mentioned much more than affect). However, in another study (Wilson et al., 2021), in-depth interviews with 36 young adults from Ghana and South Africa revealed that interviewees associated hope with concepts consistent with Snyder’s Hope Theory (e.g., efforts, capabilities, and performance) and Herth’s model of hope (e.g., community and spirituality).

Moving to student samples, Bruininks and Malle (2005) found that some students associated hope with low personal control and an inability to take action, which is somewhat at odds with Snyder’s Hope Theory. Another study revealed that for students in the sample, hope could exist in favorable *and* unfavorable conditions (Li et al., 2021). To assess the connection between lay interpretations of hope and Snyder’s theory, Tong et al. (2010) conducted four studies on students in the US and Singapore. Results indicate that the lay use of hope was associated with the agency but not the pathway component. Tong and colleagues conclude that hope can be present without believing in one’s ability to create the means to obtain the hoped-for goal.

Overall, existing research provides mixed findings about the connection between lay and scholarly interpretations of hope. There are some fundamental overlaps but also apparent inconsistencies. Note that the abovementioned studies reveal the complexities of hope and its intricate functions and connotations. However, another path is to look for the more basic parts of hope as expressed in the literal use of the word hope in everyday life.

Consider this mundane salutation: “I hope all is well on your end.” In this instance, the sender is expressing their desire for the recipient’s well-being. Whether the sender genuinely cares about the recipient’s welfare or simply follows convention, the message communicates the sender’s *wishes* that the receiver is well with no indication of the sender’s estimation of the recipient’s wellness. There are thus cases where people use the word “hope” to express their wishes, desires, or longings rather than their expectations or estimations that these wishes, desires, or longings will materialize. Essentially, “I hope” can often be interpreted simply as “I wish.”

Conversely, the meaning of hope in the sentence “My hopes for recovery are high because the surgery was successful” is not centered on the patient’s wishes for recovery but on her expectations (i.e., assessment, estimation) for recovery due to the successful medical procedure. “Hope” is thus used here to denote evaluations or expectations rather than wishes or desires. While it is evident that the patient harbors wishes and desires for good health, this wish is not the focal point of the statement, as the wish for health persists independently of the surgical outcome. “I have hope” can thus simply be interpreted as “I expect.”

What about “I hope to finish the job by the deadline”? In this example, “hope” can be understood as expressing the speaker’s *wishes* to finish the job on time, or *expectation* that the job will be completed on time, or some combination of wishes and expectations (i.e., I wish to finish the job on time, and I expect I can do it). Indeed, in numerous instances, hope encompasses a composite of wishes and expectations (Lazarus, 2013; van Zomeren et al., 2019). An intriguing but challenging aspect of hope lies in its amalgamation of wishes and expectations in an unknown proportion. Though there is a big difference between wishing for something and expecting it to materialize, determining the exact amount of wishes and expectations in the sentence “I hope to finish the job by the deadline” remains elusive.

Wishes and expectations are two distinct (though correlated) dimensions of hope (Leshem, 2017; Leshem and Halperin, 2020b; see also Sagy and Adwan, 2006; Staats, 1989; Stotland, 1969), but the colloquial use of the word uses the two interpretations interchangeably. Think about the following sentence: “I *really hope* there will be peace in the Middle East, but I have *no hope* that there will be peace in the Middle East.” Put into context, it is easy to grasp that the sentence reflects fervent wishes for peace alongside low expectations for its realization. In sum, people use hope to express the extent of their wishes for X, their expectations that X will occur, or a blend of their wishes for X and their expectation that X will occur. This ambiguity would be harmless if wishes and expectations were synonymous. But they are not (Leshem and Halperin, 2020b; Milona, 2020; Staats, 1989). One can wish for something with varying degrees of expectation that it will transpire. One can also expect something to occur with varying degrees of desire.

Researchers often use self-reported measures to gage people’s “hope” for specific outcomes (e.g., Cohen-Chen et al., 2015; Hasan-Aslih et al., 2019; van Zomeren et al., 2019). The researchers then report participants’ levels of “hope” for these outcomes. Yet, because the term is so ambiguous, it is often impossible to know if people were reporting (1) their wishes for these outcomes, (2) their expectations that these outcomes would transpire, or (3) some combination of the two. In everyday life, the ambiguity of hope can be tolerated. Indeed, one of the fascinating aspects of hope is this very ambiguous interplay between wishes and expectations. Yet, scholarship that seeks to understand hope will need a systematic approach to clarify hope’s ambiguous nature.

## The Bidimensional Model of Hope: searching for the nucleus of hope

The process leading to the Bidimensional Model of Hope was built on the need for a model that can be generalized to individual *and* collective accounts of hope and relevant to hope also outside the boundaries of personal goals and people’s actual or perceived control. To achieve this, we started by exploring the nucleus of hope, that is, hope in its narrowest form, stripped, as much as possible, of its functions and roles. The models developed by Snyder and Herth are instrumental in demonstrating how hope functions in different situations and elaborating on the process of hoping in various contexts (Feldman et al., 2023; Herth, 1991, 2005; Snyder, 2000; Snyder et al., 2005; Snyder et al., 2005). Yet, we were looking for something more basic.

Our contribution is to examine hope’s anatomical rather than functional attributes. In doing so, we seek to identify a sound starting point to help us understand hope in ways that align better with lay interpretations and are relevant to the experience of hope in social and political contexts where people’s scope of control and agency are limited. We found that the older conceptualization made by Stotland (1969), Staats (1989), and Erickson et al. (1975) are helpful because they focus on the essential elements of hope rather than how being hopeful plays out. The need to describe hope in its elemental, nuclear state corresponds with the philosophical “Standard Account” of hope, which defines hope in its narrowest, most basic form: “Hoping that P” means the “desires for P and the belief that P is possible (but not certain)” (Milona, 2020, p. 101, see also Day, 1969).

In addition to focusing on hope’s “bare” structure, we were also looking for common ground while remaining as parsimonious as possible. Looking for common ground, it seems that across scholarly *and* colloquial interpretations of hope, perhaps the most basic interpretation is that hope involves the presence of a wish for an outcome and some expectation that the outcome can be attained. Popular dictionaries also define hope as the combination of the two components. Webster’s Dictionary, for example, defines the noun hope as a “*desire* accompanied by *expectation* of or belief in fulfillment,” Oxford’s Advanced Learner’s Dictionary defines the verb hope as “to *want* something to happen and *think that it is possible*.” Understanding hope as a mental construct necessitating only two elements also serves parsimony.

At this stage, it is important to discuss our Bidimensional model vis a vis the most cited theory of hope: Snyder’s theory of agency and pathway thinking. Though our model also includes two dimensions, it differs from Snyder’s theory in two fundamental ways. First, the bidimensional model looks at hope not as a process that guides goal attainment but as a mental state that may or may not evolve into a goal-pursuing process. We believe our approach can be generalized to many situations, including those where the hoped-for outcome is located outside of the boundaries of personal goals and beyond people’s actual or perceived control (Averill et al., 1990; Bruininks and Malle, 2005). To illustrate, hope for freedom, equality, or peace could be strong and meaningful for millions worldwide. Research shows that this hope has many psychological and behavioral consequences that may or may not include the pursuit of these goals (e.g., Cohen-Chen et al., 2015; Halperin, 2016; Hasan-Aslih et al., 2020; Leshem, 2019; Rosler et al., 2017).

Second, our model describes what hope “is” but does not, in and of itself, explain how it “works.” Thus, rather than being a theory about how hope operates, our model simply suggests that people’s hope for X can be understood as a point corresponding to how much they think X is desired and how much they think X is likely to transpire. As a model describing the nuclear structure of hope, the Bidimensional Model complements rather than contradicts existing models (Herth, 1991, 2005; Snyder, 2000). As we detail later in the article, we believe the Bidimensional Model can be used as a standard reference point for future research exploring more complex properties and functions of hope.

After establishing that wishes and expectations are necessary components, we must also ensure that our search for parsimony does not overlook other elements that hope cannot do without. An intriguing approach advocated by Krafft et al. (2023) asserts that trust is the third essential element in hope. The authors define trust as the belief “in the availability of current of future internal and external resources which can facilitate the fulfillment of the hoped-for good in the faces of obstacles and setbacks” (p. 34). Because hope emerges from uncertainty, people who hope must also believe in some force, be it internal (e.g., self-agency) or external (e.g., other people, divinity), that will bring about the hoped-for outcome (see also Meirav, 2009).

Krafft and colleagues’ compelling argument that trust is an indispensable element of hope deserves close attention. Their work asserts that hope always involves trust in the current or future availability of resources and that hope is incomplete without this trust (Krafft et al., 2023). We fully agree with this statement yet argue that this trust feeds into the expectation dimension of hope and, as such, should not be understood as a standalone element. To explain our argument, it is first necessary to ascertain that hope is a purely subjective mental construct and that people’s wishes and expectations are subjectively evaluated. When people appraise the desirability of an outcome, they may rely on different cues, information sources, and beliefs and, like all subjective processing, be susceptible to bias. People’s expectations of fulfillment are also subjective. When people assess the likelihood of an outcome, they may also rely on different cues, beliefs, or sources of information that are then subjectively processed and may also be influenced by biases (Fiske, 2010).

We claim that trust is one of these cues, information, and beliefs that feed into the expectation dimension. When one trusts that external resources like family members, the medical team, or God will help them recover from severe illness, one is likely to incorporate this trust into the subjective evaluation of the chances for recovery. When one trusts internal resources like personal strength and determination, one inadvertently weighs this into one’s estimation of recovery. Indeed, in times of hardship and calamity, when “objective evidence” points to unfavorable outcomes, people’s expectation that their goals will be attained may be almost exclusively driven by their trust in external or internal forces that will facilitate attaining a goal. In other words, trust in the availability of resources is a crucial ingredient of hope, but we see it as an ingredient included within the expectation dimension.

## The Bidimensional Model of Hope: wishes and expectations as orthogonal dimensions

Up to this stage, our main points were that reducing hope to its core components will always leave us with two elements, namely, wishes and expectations, and that looking at hope as the combination of these two elements does not seriously infringe scholarly or colloquial interpretations of the concept. The next natural question is how these two components compile into “hope.” Do wishes and expectations add up to create hope? Is hope a product of their multiplication? Let us examine these options.

One approach used in Snyder’s Trait, State, and Domain Hope Scales and Herth’s Hope Scale is to calculate the total hope score by summing the scores of each item across all factors (Herth, 1991; Snyder, 2000). However, determining the levels of hope through aggregation might prove problematic when one factor equals zero. Because, according to these theories, each factor is essential, hope should logically be zero when one factor is null, irrespective of the magnitude of the other factors. Yet summing (or averaging) the scores when one factor equals zero and the other more than zero will always give a positive score. Going back to our bidimensional model, in scenarios where the wish is absent but some level of expectation persists, combining (or averaging) the wishes and expectations results in a numerical value greater than zero. Nonetheless, hope does not exist when we do not wish for an outcome. The conclusion is that hope cannot be the aggregation of wishes and expectations.

Multiplying wishes and expectations (see Staats, 1989) solves this problem and is in line with work on motivation that uses the product of the perceived value of a goal and the perceived expectancy to achieve a goal as a predictor of behaviors pursuing the goal (Forster et al., 2007; Kruglanski et al., 2015). The multiplication approach also resonates with economic frameworks of expected utility (Schoemaker, 1982), wherein the value of a prospect is multiplied by its likelihood of occurrence. However, defining hope as the product of wishes and expectations poses two fundamental challenges. The first pertains to the assumption of equal weighting between wishes and expectations in the multiplication process. However, the weight of each dimension—namely, its contribution to hope—is contingent upon contextual factors. In certain scenarios, hope may hinge more prominently on one’s strong wishes for an outcome rather than the expectations surrounding its attainment, whereas in other instances, the influence of expectations may outweigh that of wishes.

The second challenge arises from the non-interchangeability of wishes and expectations. For instance, the hopes harbored by an individual with low wishes but high expectations differ qualitatively from those of another individual with high wishes but low expectations regarding the same outcome. The former individual exhibits modest enthusiasm toward the outcome yet perceives it as feasible, whereas the latter holds strong wishes for the outcome but perceives its attainment as improbable. Despite yielding identical multiplication products, their hopes for the outcome possess distinct qualitative attributes.

**Figure 1:**
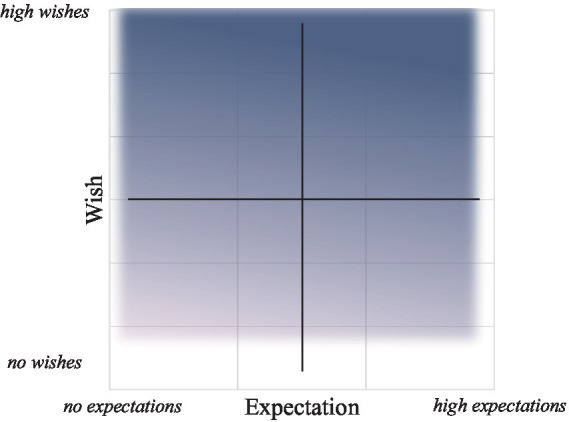
The Bidimensional Model of Hope.

How can we define hope as a construct combining wishes and expectations if we cannot sum or multiply them? To tackle this puzzle, we follow Fiske’s (2018) and Fiske et al. (2007) approach to looking at a sociopsychological construct made of two components as a bidimensional construct. Instead of aggregating or multiplying two components to create a single value, the bidimensional approach looks at the sociopsychological construct as a point on a bidimensional plane corresponding to two values. In our case, the Bidimensional Model of Hope (see Figure 1) maps hope on two orthogonal dimensions, with one dimension being the wish for the outcome and the other being the expectations of its materialization. The levels of hope (from non-existent to very high) could be found anywhere on this bidimensional plane. As we exemplify in the following paragraphs, the bidimensional model provides much-needed nuances to understanding hope in various contexts.

## Conceptual utility of the Bidimensional Model of Hope

The conceptual utility of the model is exemplified by a simple examination of the bidimensional plane presented in Figure 1 (divided into quarters to facilitate the exploration). First, hope is virtually non-existent when wishes and expectations approach zero (located at the bottom-left corner, beyond the shaded region).^3^ Progressing upward and toward the right, we transition into the shaded region, denoting hope across its myriad combinations and intensities. Hope correspondingly increases with the rise of wishes and expectations (i.e., as we approach the upper-right corner). At its zenith, hope is highest in the upper-right corner when both wishes and expectations are greatest. However, when expectations ascend to the point of certainty (very high expectations at the extreme right side of the figure), hope becomes irrelevant. In situations of certainty, we depart from the realm of hope, as there is no inclination to hope for events deemed inevitable.

Examining the shaded area, it becomes evident that hope levels range from low in the lower-left section to high in the upper-right section. However, the remaining two shaded areas warrant special attention. Firstly, let us consider the lower-right quadrant, where wishes for an outcome are low to moderate, and expectations are high. This quadrant characterizes situations where individuals exhibit lukewarm enthusiasm toward an outcome while maintaining a belief in its achievability. This scenario might align with the concept of optimism. Compared to hope, optimism often pertains to objectives perceived as less significant but deemed more feasible (Bruininks and Malle, 2005).

The upper-left corner is the most interesting section as it represents situations where the wishes are high but expectations are low. These situations arise when outcomes are deemed essential but unlikely. A severely ill person fighting for her health, a historically marginalized community struggling for social mobility, and societies mired in decades of violent conflict striving for peace are all examples of situations where certain outcomes are desperately needed, but their chances to materialize might be exceptionally slim.

Located in the upper-left corner, these intense experiences of hope (for health, social mobility, or peace) can be strong and meaningful, but they are driven by strong desires and wishes, not by the expectations of fulfillment. There are indeed many times when, despite the small likelihood of attainment, people’s hopes are unwavering. People’s determination to fight for democracy and freedom in totalitarian and oppressive regimes or their struggles for peace amid violent conflict are driven by unshaken wishes for social change *despite* the low odds of attainment. The common phrase, “hope in the absence of hope,” is thus not a paradox but a way to express the presence of desires, dreams, and aspirations when the likelihood of fulfillment is scant. Thinkers and public figures also focused on this type of hope, where we desire something badly despite our understanding that the chances of achievement are meager. As Vaclav Havel said: “*The more unpropitious the situation in which we demonstrate hope, the deeper that hope is*.”

The argument that hope is a bidimensional construct is further validated in Exploratory Factor Analysis. For example, in a study on Jewish-Israelis and Palestinians’ hope for peace, items measuring participants’ wishes for peace loaded well on one factor (>0.62), while items gaging participants’ expectations for peace loaded well on a second factor (>0.65). No cross-loadings over 0.2 were observed (Leshem and Halperin, 2020b). It is important to note that the orthogonal nature of the dimensions does not imply that they are not correlated. Our studies reveal, for example, that the correlation between wishes and expectations for peace among people mired in intractable conflicts (in this case, Jewish Israelis and Palestinians from the Occupied Territories) is significant and ranges between 0.18 and 0.44 (Leshem, 2017, 2019; Leshem and Halperin, 2023). Generally speaking, the correlations between the dimensions may vary depending on numerous factors, including the target of hope, the context, the population studied, and so on.

The Bidimensional Model of Hope could also help in solving the puzzle of the opposite of hope. In some literature, despair is considered the antonym of hope (Fletcher, 1999; Halperin, 2016; Nesse, 1999). Lazarus explicitly contrasts hope and despair, with the former being a vital coping mechanism against the latter (Lazarus, 1999). Lazarus positions hope and despair as opposite reactions to different perceptions of reality (hope is associated with perceiving a positive future and despair with perceiving a negative future) and as opposites in terms of virtue, with hope being constructive and despair destructive to human well-being.

Other research mentions fear as the opposite of hope (Jarymowicz and Bar-Tal, 2006; Roseman, 1991). This claim is also plausible as hope involves envisioning a positive future while fear involves envisioning an adverse future. Fear and hope can also be regarded as opposites in terms of their psychological mechanisms (Bar-Tal, 2001). Fear is much more automatic and often requires little cognitive resources (LeDoux, 1998), whereas hope involves taxing cognitive activity, including creativity and flexibility (Snyder, 1994). Halperin et al. (2008) found that in the context of conflict, the two constructs are negatively correlated (*r* = −22 *p* < 0.001). In another study conducted in the context of conflict, fear and hope had opposite effects on information-seeking (Cohen-Chen et al., 2014). Hope directed people to seek information about opportunities for peace, while fear inclines people to acquire information that rejects these opportunities.

Fear and despair are not synonymous. So, which one is hope’s opposite? The bidimensional approach quickly solves this dilemma. Quite simply, both are the opposites of hope (see also Blöser, 2020; Day, 1969). Despair is elicited when an outcome is appraised as highly desirable, but expectations of its realization are negative. Simply put, we wish very much but know attainment is impossible. The more we wish for something and the more we acknowledge the impossibility of attainment, the greater the despair. On the bidimensional plane, despair is located to the left of the Y-axis (denoting negative expectations) and above the X-axis (denoting a positive wish). Fear, on the other hand, is elicited when probabilities are appraised as high, but the outcome is adverse. We do not want something malign or hurtful to happen, but we believe it will. The more adverse the event and the more likely it is, the more we fear it. On the bidimensional plane, fear is placed to the right of the Y-axis (denoting positive expectations) but below the X-axis (denoting a negative wish). Understanding hope as a bidimensional construct helps in its conceptualization. As the following section shows, the bidimensional approach also has methodological advantages.

## Methodological utility of the Bidimensional Model of Hope

Transitioning the bidimensional model from conceptualization to operationalization is relatively straightforward. To determine the levels of hope for X, we need to determine the degree to which people wish for X and the degree to which people expect X to materialize. For example, using self-reported items, we can ask how much, on a scale from 1 to 6, one *wishes* for a certain outcome and then ask how much one *expects* the outcomes to transpire (see Erickson et al., 1975; Sagy and Adwan, 2006; Staats, 1989). We then plot the answers on a bidimensional plane to determine the hopes for that outcome. Since 2017, we and our colleagues have been operationalizing hope based on the bidimensional model described above (Hasler et al., 2023; Leshem, 2017, 2019; Leshem et al., 2023a; Leshem et al., 2023b; Ushomirsky et al., 2023; Leshem and Halperin, 2020a, 2020b, 2023; Shani et al., in press; Ushomirsky et al., 2023).

Our experience demonstrates the usefulness of the bidimensional model. For instance, instead of comparing people’s “hopes” for an outcome, researchers can use the bidimensional approach to explore the levels of wishes for the outcomes and conduct a separate investigation comparing people’s expectations for the outcomes. In one study, for example, we show that Israelis’ wish for peace decreases as the definition of peace becomes more concrete, but the levels of expectations for peace remain the same regardless of the concreteness of the definition (Leshem, 2017). Separately measuring wishes and expectations can thus help in better understanding the dynamics of hope across contexts.

Moreover, researchers can conduct a much more nuanced study of hope’s correlates using the bidimensional approach. For example, in the study of hope in political contexts, political efficacy, that is, people’s belief in their ability to influence political outcomes, can be easily conflated with the concept of hope. Yet, a study conducted in the context of conflict shows that political efficacy is associated only with the expectation dimension of hope, not the wish dimension (Leshem and Halperin, 2020a). In the same study, we show that the extent to which people feel comfortable with uncertainty is correlated with the wish dimension and not with the expectation dimension. It seems that people who are more comfortable with uncertainty and unpredictability allow themselves to wish for certain political outcomes more than those who feel uncomfortable with uncertainty, but this trait has no association with the expectation dimension.

Furthermore, the bidimensional approach can be used to test new hypotheses in both quantitative and qualitative research designs. In one study, we analyzed speeches made by Israeli and Palestinian leaders speaking at the UN General Assembly to test whether the power disparity between the groups is associated with the frequency of leaders’ expressions of hope for peace (Ushomirsky et al., 2023). As we postulated, across 46 speeches made by 13 different speakers, leaders of the low-power group (in this case, Palestinians) expressed more hope for peace compared to leaders’ of the high-power group (in this case, Israelis), but only on the wish dimension. It appears that Palestinian leaders expressed more wishes (i.e., desires, aspirations) for peace than Israeli leaders but that the expectations for peace (belief in the possibility of peace) were equally low regardless of the speaker’s nationality.

In another study, this time qualitative, we interviewed peace activists and analyzed the way they used the word hope when interviewed (Leshem et al., 2023a). Though hope was used to signify both dimensions, we noticed that when explaining their motivation for activism, hope was used to signify wishes rather than expectations. When we explicitly asked interviewees why this was the case, they explained that, for them, hope was more about the desires and aspirations for peace and not so much about the chances that peace will materialize.

These are just some examples of how the bidimensional model can be used to expand our understanding of hope. The common response we receive from academic and non-academic audiences is that the model is very intuitive. We believe that the simplicity and intuitiveness of the bidimensional model are what makes it versatile and applicable across contexts and disciplines. Regardless of the target of hope and whether it is practiced on the individual or societal level, the bidimensional approach facilitates and simplifies hope research and enriches the study of hope across disciplines. In the following discussion, we summarize our claims highlight several promising directions for further research, expanding the ways and contexts in which the Bidimensional Model of Hope can be applied.

## Discussion

The article introduces the Bidimensional Model of Hope and its utility in explaining and measuring hope. More broadly, acknowledging that hope is comprised of some interplay between wishes and expectations and that these two constructs are not interchangeable begs that we try to examine them separately. In other words, if we want to understand people’s hope for some outcome, we must understand the intensity of people’s wishes and desires for this outcome separately from their expectations and assessments that these outcomes will transpire.

That said, a question arises as to why the bidimensional approach was not proposed and employed throughout the history of hope research. We propose that at least two factors hindered the development of the Bidimensional Model of Hope. Firstly, “hope” is a nebulous term, and this inherent ambiguity is difficult to eliminate. It could certainly be the case that the colloquial use of “hope” misled scholarly inquiries, obscuring the distinction between the two dimensions. Secondly, even hope scholars who did recognize the two components of hope (Sagy and Adwan, 2006; Staats, 1989; Stotland, 1969) did not propose a coherent model that connects them. Without such a model, conceptualizing hope as the combination of wishing and expecting remained a scattered thought, not a robust theoretical and empirical framework.

The bidimensional approach is still in its infancy and, as such, requires further validation, exploration, and implementation. We also do not suggest that the bidimensional model is the ultimate or only way to understand hope. Instead, we propose that understanding hope as a bidimensional mental construct provides a sound and bountiful starting point for hope research. First, discerning between the two dimensions of hope opens the door for the big questions about the role of hope in our lives. Looking outward, we can ask: Which dimension is more important when it comes to human advancement? People’s wishes for a certain outcome or their belief that they have a chance to materialize it? Looking inward, we can try to understand which dimension is more central as an individual coping mechanism in times of adversity. More generally, we can ask questions about the normative and psychological consequences stemming from the innate blurriness between the dimensions and inquire whether deliberately distinguishing between wishing and expecting has normative or psychological benefits.

In addition to these and other “big questions,” some more nuanced directions could be suggested. For example, separating between wishes and expectations enables us to explore the *gap* between the two dimensions. Naturally, when wishes and expectations are congruent, the levels of frustration are low. This could be the case when people are indifferent toward a certain outcome and assess its feasibility as low or when people have high desires for an outcome, believing its materialization is nearing. However, when wishes and expectations are incongruent, and specifically, when wishes are high but expectations are low (upper left-corner of the bidimensional plane), frustration is likely to rise. This hope gap (i.e., the gap between wishes and expectations) can then be explored as a predictor in inferential models.

Apart from the gap, researchers could also investigate the correlation between wishes and expectations and trace the conditions that affect the correlations between the dimensions. Studies that identify situations where wishes and expectations are strongly vs. weakly correlated could be eye-opening in many research fields in the social sciences. For example, we could examine the dynamics of the correlation between wishes and expectations across time, holding the population and the target of hope constant. Holding the target of hope constant (for example, physical well-being), we could also detect personalities exhibiting high vs. low correlations between the dimensions.

Future research utilizing the Bidimensional Model of Hope could further test the relative predicting power of wishing vs. expecting in social and political contexts. For instance, it could be revealing to know which dimension is more predictive of peoples’ preferences for certain policies or of peoples’ willingness to participate in collective action; is it their wishes for certain political outcomes or their expectations that these outcomes can materialize Examining the relative predicting power of each dimension could also be instructive in studies on the role of hope in the context of personal academic and athletic achievements, personal well-being, and physical health. Experimental research could reveal the causal effect of specific cues on each of the dimensions of hope and then test how potential changes in each dimension affected attitudes, emotions, or behaviors.

Another open avenue is the question of interaction. Do the dimensions interact to elicit “hope”? Are there boundary conditions such that one dimension is activated only when the other is within a certain range? To that extent, we cannot assume the potential interaction is linear (see Bury et al., 2018 for a similar approach). There are indeed many paths to investigate the question of interaction, which will surely bring us closer to understanding how hope “works.”

## Conclusion

Much work is still needed to develop and test the utility of the Bidimensional Model of Hope. We encourage scholars of hope, as well as other researchers across disciplines, to apply the model in their studies, test its effectiveness, and compare its robustness with other approaches. These attempts will surely benefit our understanding of hope and how it functions on the individual and societal levels.

As we are writing this paper, Israelis and Palestinians are experiencing what is probably the worst episode in the conflict’s history. Yet, long before the current war, one of the most widely held beliefs among Israelis and Palestinians is that the conflict can never be resolved (Bar-Tal, 2013). The complete lack of hope for peace (in this case, in the expectation dimension) quenches any motivation to work for conciliation and thus further sustains the conflict. Our goal is to understand how hope for peace can be formed in such dire circumstances and how it can, in turn, promote the materialization of peace for the sake of all residents of the region.

## Footnotes

^1^We thank the anonymous reviewer for pointing to Herth’s important work.

^2^Rose notes that collapsing pathway thinking and agency may sometimes provide a better-fitted model when translated into non-English languages (Rose, 2022).

^3^Note that not wishing for X does not necessarily mean wishing for the opposite. Similarly, not expecting X to materialize, does not necessarily mean expecting the opposite to materialize.
